# Soil properties changes earthworm diversity indices in different agro-ecosystem

**DOI:** 10.1186/s12898-020-00296-5

**Published:** 2020-05-07

**Authors:** Sharanpreet Singh, Ayushi Sharma, Kiran Khajuria, Jaswinder Singh, Adarsh Pal Vig

**Affiliations:** 1 Department of Botanical & Environmental Sciences, Guru Nanak Dev University, Amritsar, Punjab India; 2 Post Graduate Department of Zoology, Khalsa College, Amritsar, Punjab India

**Keywords:** Abundance, Earthworms, Land use pattern, Soil variables, Principal component analysis

## Abstract

**Background:**

Earthworm communities are generally very sensitive to physico-chemical properties of the soil in different agro-ecosystem i.e. cultivated or non-cultivated which directly or indirectly influence the earthworm survival. The difference in physico-chemical properties of soil at different sites contributed to the formation of population patches for earthworm species. Understanding the physico-chemical properties of soil at a particular site could facilitate the prediction of earthworm species at that site. The objective of the present study was to investigate the diversity, abundance, and distribution of earthworms in cultivated and non-cultivated agroecosystems and their physico-chemical properties affecting the earthworm diversity and abundance.

**Results:**

Total 10 species of earthworms i.e. *Amynthas alexandri, Amynthas morrisi, Eutyphoeus incommodus, Eutyphoeus waltoni, Metaphire birmanica, Metaphire houlleti, Metaphire posthuma, Octochaetona beatrix, Perionyx excavatus,* and *Polypheretima elongata,* were reported. Out of all the reported species, *Metaphire posthuma* was found to be the most abundant earthworm species in both cultivated and non-cultivated agroecosystems with the occurrence at 56.81% sites. The Shannon-Wiener index (H), Margalef species richness index (D_Mg_) and Pielou species evenness (E) was ranged from 0 to 0.86, 0 to 0.64 and 0.78 to 1 respectively. The principal component analysis resulted in four principal components i.e. PC1, PC2, PC3 and PC4 which contributing variance (%) of 22.96, 19.37, 14.23 and 10.10 respectively. The principal component analysis also showed that physico-chemical parameters of soil such as EC, pH, TDS, texture, OC, moisture, etc. play a critical role in earthworm distribution.

**Conclusion:**

The conventional farming system has a negative effect on the earthworm diversity in the soil while the physico-chemical properties of soil also have a determinant effect on the same. Earthworms abundance in the present study have significant direct relation with soil properties at a particular site and vice versa. The diversity indices also change due to the conventional farming system which directly affects the earthworm abundance.

## Background

In agro-ecosystems, earthworm communities are generally very sensitive to physico-chemical properties of the soil which directly or indirectly influence the availability of resources for earthworm survival [1]. The soil structure and its pore size also play a key role in the distribution of the earthworms within the soil and a minute changes in the same can adversely affect the earthworm’s community structure [2]. The richness and diversity of earthworm species were always observed higher in the undisturbed land as compared to disturbed land and likely to increase under favorable soil conditions [3, 4]. In undisturbed land, soil characteristics such as soil quality [5]; pH [6]; moisture [7], as well as soil organic matter [8], affect the diversity and abundance of earthworm’s species. On the other hand, disturbed land such as the agricultural field, application of fertilizer [9]; pesticides [10]; tillage [11] and soil organization [12] have a strongest effect on earthworm distribution.

Earthworms act as an ecosystem engineer by modifying soil structure and its properties [13–15]. It also change the porosity of the soil by regulating the proportion of organic matter breakdown and nutrient release [16]. The difference in physico-chemical properties of soil at different sites contributed to the formation of population patches for earthworm species. By knowing the physico-chemical properties of soil at a particular site could facilitate the prediction of earthworm species at that site [17]. It is also important to quantify the spatial distribution of earthworms at different agroecosystems in order to understand the effect of abiotic soil processes and to link earthworm abundance to the spatial distribution of macropores in the soil. The information collected on type of earthworm species and their abundance at different habitats may also provide useful information on the efficiency and strength of that ecosystem. Besides the soil physico-chemical properties, the types of the agro-ecosystem selected at various site i.e. cultivated or non-cultivated are also play an important role in earthworms abundance and distribution.

The objective of the present study was to investigate the diversity, abundance, and distribution of earthworms in cultivated and non-cultivated agroecosystems and their physico-chemical properties affecting the earthworm diversity and abundance.

## Results

### Earthworm communities and their diversity indices

**Fig. 1:**
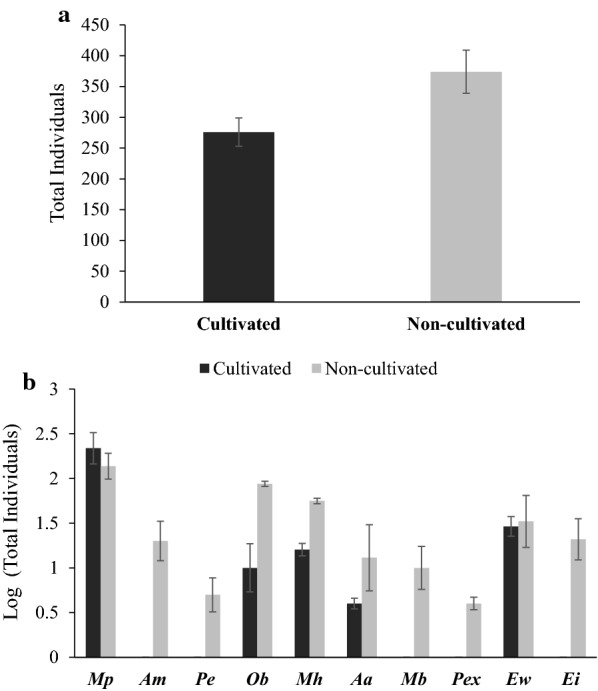
The total number of individuals sampled (**a**) and total individuals sampled (log transformed) in each earthworm species (**b**) in cultivated and non-cultivated sampling sites

We have explored the 44 different sites under cultivated and non-cultivated agroecosystem (Additional file 1: Table S1) under which total 10 species of the earthworms viz. *Amynthas alexandri, Amynthas morrisi, Eutyphoeus incommodus, Eutyphoeus waltoni, Metaphire birmanica, Metaphire houlleti, Metaphire posthuma, Octochaetona beatrix, Perionyx excavatus* and *Polypheretima elongata* belonging to two families and six genera were reported. Out of these, 7 species belong to family Megascolecidae (*M. posthuma, A. morrisi, A. alexandri, M. houlleti, Polypheretima. elongata, Perionyx excavatus* and *M. birmanica)* and three species belong to family Octochaetidae (*O. beatrix*, *E. waltoni* and *E. incommodus*). The distribution of above said species along with their ecological category and diversity indices at each sampling site is given in Table 1. The non-cultivated agroecosystem has high earthworm abundance as compared to cultivated agroecosystem (Fig. 1a). The cultivated and non-cultivated agroecosystem has 5 and 10 earthworm species respectively. The abundance pattern in cultivated and non-cultivated agro-ecosystem was in the order of *M. posthuma* > *E. waltoni* > *M. houlleti* > *O. beatrix *> *A. alexandri* and *M. posthuma* > *O. beatrix *>* M. houlleti* > *E. waltoni* > *E. incommodus* > *A. morrisi* > *A. alexandri* > *M. birmanica* > *Polypheretima elongata* > *Perionyx excavatus* respectively (Fig. 1b). Out of all the reported species, *M. posthuma* was found to be the most abundant earthworm species in both cultivated as well non-cultivated agroecosystems with the occurrence at 56.81% sites. The Shannon-Wiener diversity index (H), Margalef species richness index (D_Mg_) and Pielou species evenness (E) were ranged from 0 to 0.86, 0 to 0.64 and 0.78 to 1 respectively (Table 1). The different values of Shannon-Wiener diversity, species richness and species evenness index for the same number of earthworm individual at few sites were due to the difference in the number of earthworm species.

**Table 1:** The earthworm abundance (species wise) and their diversity indices in different sampling sites

Sites	Individuals/m^2 *^	*M. posthuma* (Endogeic)	*A. morrisi* (Anecic)	*A. alexandri* (Anecic)	*E. waltoni* (Anecic)	*E. incommodus* (Anecic)	*M. houlleti* (Endogeic)	*O. beatrix* (Endogeic)	*P. excavatus* (Epigeic)	*M. birmanica* (Endogeic)	*P. elongata* (Endogeic)	Shannon Wiener Diversity Index (H’)**	Species Richness (D_Mg_)**	Species Evenness (E)**
Spot 1	15 (*Mp*)	++	−	−	−	−	−	−	−	−	−	0	0	1
Spot 2	15 (4 *Ew*, 11 *Ei*)	−	−	−	+	++	−	−	−	−	−	0.57	0.36	0.89
Spot 3	10 (*Ei*)	−	−	−	−	+	−	−	−	−	−	0	0	1
Spot 4	14 (*Am*)	−	++	−	−	−	−	−	−	−	−	0	0	1
Spot 5	17 (*Mp*)	++	−	−	−	−	−	−	−	−	−	0	0	1
Spot 6	12 (*Mp*)	++	−	−	−	−	−	−	−	−	−	0	0	1
Spot 7	9 (*Mp*)	++	−	−	−	−	−	−	−	−	−	0	0	1
Spot 8	16 (*Mp*)	++	−	−	−	−	−	−	−	−	−	0	0	1
Spot 9	15 (*Mp*)	++	−	−	−	−	−	−	−	−	−	0	0	1
Spot 10	18 (5 *Mh*, 13 *Mp*)	++	−	−	−	−	+	−	−	−	−	0.59	0.34	0.90
Spot 11	11 (*Mp*)	++	−	−	−	−	−	−	−	−	−	0	0	1
Spot 12	17 (*Mp*)	++	−	−	−	−	−	−	−	−	−	0	0	1
Spot 13	12 (*Mp*)	++	−	−	−	−	−	−	−	−	−	0	0	1
Spot 14	15 (*Mp*)	++	−	−	−	−	−	−	−	−	−	0	0	1
Spot 15	15 (12 *Mp*, 3 *Ew*)	++	−	−	+	−	−	−	−	−	−	0.50	0.36	0.82
Spot 16	13 (*Mp*)	++	−	−	−	−	−	−	−	−	−	0	0	1
Spot 17	12 (9 *Mp*, 3 *Aa*)	++	−	+	−	−	−	−	−	−	−	0.56	0.40	0.87
Spot 18	16 (*Mp*)	++	−	−	−	−	−	−	−	−	−	0	0	1
Spot 19	17 (13 *Mp*, 4 *Aa*)	++	−	+	−	−	−	−	−	−	−	0.54	0.35	0.86
Spot 20	12 (*Ob*)	−	−	−	−	−	−	++	−	−	−	0	0	1
Spot 21	13 (7 *Ob*, 6 Mh)	−	−	−	−	−	++	++	−	−	−	0.69	0.38	0.99
Spot 22	16 (10 *Ob*, 6 Ew)	−	−	−	+	−	−	++	−	−	−	0.66	0.36	0.96
Spot 23	16 (12 *Ob*, 4 *Mb*)	−	−	−	−	−	−	++	−	+	−	0.56	0.36	0.87
Spot 24	13 (*Mh*)	−	−	−	−	−	++	−	−	−	−	0	0	1
Spot 25	17 (*Mh*)	−	−	−	−	−	++	−	−	−	−	0	0	1
Spot 26	14 (8 *Ob*, 6 *Ew*)	−	−	−	+	−	−	++	−	−	−	0.68	0.37	0.98
Spot 27	11 (*Ew*)	−	−	−	++	−	−	−	−	−	−	0	0	1
Spot 28	13 (*Ob*)	−	−	−	−	−	−	++	−	−	−	0	0	1
Spot 29	9 (*Ew*)	−	−	−	++	−	−	−	−	−	−	0	0	1
Spot 30	12 (8 *Mh*, 4 *Pex*)	−	−	−	−	−	++	−	+	−	−	0.63	0.40	0.94
Spot 31	14 (*Ob*)	−	−	−	−	−	−	++	−	−	−	0	0	1
Spot 32	16 (*Mh*)	−	−	−	−	−	++	−	−	−	−	0	0	1
Spot 33	12 (*Ob*)	−	−	−	−	−	−	++	−	−	−	0	0	1
Spot 34	16 (9 *Ob*, 7 *Mh*)	−	−	−	−	−	+	++	−	−	−	0.68	0.36	0.99
Spot 35	11 (*Ew*)	−	−	−	++	−	−	−	−	−	−	0	0	1
Spot 36	18 (13 *Mp*, 5 *Pe*)	++	−	−	−	−	−	−	−	−	+	0.59	0.34	0.90
Spot 37	21 (13 *Mp*, 8 *Aa*)	++	−	+	−	−	−	−	−	−	−	0.66	0.32	0.97
Spot 38	16 (*Mp*)	++	−	−	−	−	−	−	−	−	−	0	0	1
Spot 39	18 (*Mp*)	++	−	−	−	−	−	−	−	−	−	0	0	1
Spot 40	22 (14 *Mp*, 6 *Am*, 2 *Aa*)	++	+	+	−	−	−	−	−	−	−	0.86	0.64	0.78
Spot 41	22 (16 *Mp*, 6 *Mb*)	++	−	−	−	−	−	−	−	+	−	0.58	0.32	0.89
Spot 42	18 (*Mp*)	++	−	−	−	−	−	−	−	−	−	0	0	1
Spot 43	16 (*Mp*)	++	−	−	−	−	−	−	−	−	−	0	0	1
Spot 44	15 (*Mp*)	++	−	−	−	−	−	−	−	−	−	0	0	1

+ = Abundance (Species have 1 to 50% population); ++ = Most abundance (Species have more than 50% population); − = Absent (No species reported)

* *Mp: Metaphire posthuma; Ew: Eutyphoeus waltoni; Ei: Eutyphoeus incommodus; Am: Amynthas morrisi; Mh: Metaphire houlleti; Aa: Amynthas alexandri; Ob: Octochaetona beatrix; Mb: Metaphire birmanica*

*Pex: Perionyx excavatus; Pe: Polypheretima elongata*

** Shannon–Wiener diversity index, Species richness and Species evenness was applied only on adult earthworms

### Relation between physico-chemical properties of soil and earthworm abundance

The physico-chemical analysis of soils of all sampling sites is given in Table 2. The texture of soil was found to be loam to sandy loam at maximum sites with sand (%), silt (%) and clay (%) were in the range of 43.28–81.47, 5.34–44.21 and 6.54–27.61 respectively. The moisture content of the soil at each sampling site was more than 45%. The earthworm species were distributed in the soil moisture range from 48–97%, pH from 5.96–8.65, EC from 27.3–897.5 µS, TDS from 27.45 to 166.5 mg/L, OC from 1.01–9.53%, N from 0.12–2.68 g/Kg, P from 0.06–0.26 g/Kg, K from 0.97 to 7.45 g/Kg, Ca from 1.18–107.34 g/Kg and Na from 0.44 to 1.45 g/Kg.

**Table 2:** The physico-chemical properties of soil samples collected from each earthworm sampling site

Sample sites	Soil Texture	Sand (%)	Silt (%)	Clay (%)	Moisture (%)	pH	EC (µS)	TDS (mg/L)	OC (%)	N (g/Kg)	P (g/Kg)	K (g/Kg)	Ca (g/Kg)	Na (g/Kg)
Spot 1	Sandy Loam	65.23 ± 0.21	22.41 ± 0.27	12.36 ± 1.71	82 ± 0.18	8.50 ± 0.58	129 ± 2.38	93.05 ± 0.85	1.45 ± 0.24	1.27 ± 0.05	0.11 ± 0.01	2.96 ± 0.014	10.70 ± 0.67	0.63 ± 0.02
Spot 2	Loam	45.14 ± 0.21	38.91 ± 0.14	15.95 ± 2.35	79 ± 0.83	8.65 ± 0.95	80.85 ± 1.27	57.45 ± 1.27	2.77 ± 0.65	1.31 ± 0.09	0.21 ± 0.01	2.38 ± 0.03	8.88 ± 0.98	0.94 ± 0.01
Spot 3	Sandy Loam	81.47 ± 0.08	8.46 ± 0.03	10.07 ± 1.1	84 ± 0.09	7.14 ± 0.19	897.5 ± 9.38	89.8 ± 5.84	3.70 ± 0.85	1.35 ± 0.04	0.21 ± 0.02	7.45 ± 0.16	18.64 ± 1.84	0.74 ± 0.03
Spot 4	Sandy Loam	69.49 ± 0.01	20.31 ± 0.02	10.2 ± 2.88	89 ± 1.76	7.98 ± 0.93	118.15 ± 4.23	83.95 ± 1.86	3.08 ± 0.18	1.05 ± 0.02	0.08 ± 0.01	1.61 ± 0.09	10.20 ± 0.83	0.75 ± 0.03
Spot 5	Loam	51.24 ± 0.01	30.27 ± 0.51	18.49 ± 0.44	87 ± 0.13	8.12 ± 0.37	129.35 ± 2.67	91.70 ± 2.54	6.57 ± 0.46	1.17 ± 0.03	0.18 ± 0.01	1.52 ± 0.21	6.24 ± 0.98	0.85 ± 0.02
Spot 6	Sandy Loam	69.17 ± 0.31	16.84 ± 0.27	13.99 ± 3.54	91 ± 0.15	8.12 ± 0.83	69.75 ± 1.34	49.55 ± 0.96	3.54 ± 0.67	1.31 ± 0.14	0.17 ± 0.01	1.28 ± 0.04	9.52 ± 0.34	0.65 ± 0.05
Spot 7	Loam	43.87 ± 0.2	36.41 ± 0.31	19.72 ± 1.34	85 ± 0.11	8.22 ± 0.71	140.25 ± 2.27	99.95 ± 1.38	7.00 ± 0.49	0.28 ± 0.02	0.11 ± 0.03	2.23 ± 0.14	18.02 ± 1.38	0.54 ± 0.01
Sport 8	Loam	45.96 ± 0.28	33.29 ± 0.11	20.75 ± 3.15	76 ± 1.32	8.52 ± 0.25	111.25 ± 3.19	78.90 ± 2.74	3.59 ± 0.31	0.37 ± 0.01	0.15 ± 0.01	2.22 ± 0.31	10.08 ± 1.82	0.87 ± 0.03
Spot 9	Sandy Loam	59.46 ± 0.32	24.47 ± 0.45	16.07 ± 0.56	95 ± 0.87	8.57 ± 0.39	88.60 ± 1.38	63.55 ± 1.62	4.25 ± 0.85	0.23 ± 0.01	0.08 ± 0.01	1.62 ± 0.08	19.41 ± 0.92	0.69 ± 0.05
Spot 10	Sandy Loam	53.14 ± 0.26	32.41 ± 0.65	14.45 ± 0.55	93 ± 0.38	7.09 ± 0.42	38.60 ± 1.84	27.45 ± 0.25	3.83 ± 0.67	0.26 ± 0.03	0.06 ± 0.01	1.56 ± 0.17	12.92 ± 0.37	0.59 ± 0.01
Spot 11	Sandy Loam	57.47 ± 0.24	24.29 ± 0.3	18.24 ± 0.1	96 ± 0.64	8.14 ± 0.83	143.85 ± 2.62	102.50 ± 1.24	2.08 ± 0.16	2.68 ± 0.12	0.10 ± 0.01	2.16 ± 0.31	107.34 ± 2.47	0.68 ± 0.03
Spot 12	Sandy Loam	55.16 ± 0.15	26.08 ± 0.2	18.76 ± 0.64	97 ± 0.81	7.15 ± 0.16	61.20 ± 2.47	43.50 ± 1.37	1.82 ± 0.27	0.29 ± 0.02	0.17 ± 0.01	2.43 ± 0.41	8.22 ± 0.34	0.44 ± 0.02
Sport 13	Loam	50.89 ± 0.19	29.98 ± 0.75	19.13 ± 0.05	96 ± 0.58	7.68 ± 0.73	134.05 ± 4.31	94.80 ± 2.92	9.53 ± 0.39	0.36 ± 0.01	0.19 ± 0.03	3.60 ± 0.19	17.97 ± 0.93	0.64 ± 0.02
Spot 14	Sandy Loam	63.24 ± 0.02	22.94 ± 0.75	13.82 ± 1.04	83 ± 0.54	7.84 ± 0.42	50.05 ± 1.35	35.85 ± 1.76	5.22 ± .49	0.51 ± 0.01	0.12 ± 0.03	2.10 ± 0.08	12.42 ± 1.08	0.60 ± 0.02
Spot 15	Sandy Loam	71.38 ± 0.6	15.77 ± 1.05	12.85 ± 0.18	93 ± 0.52	8.13 ± 0.16	145.50 ± 1.76	102.50 ± 0.98	3.77 ± 0.67	0.36 ± 0.03	0.10 ± 0.01	0.98 ± 0.01	16.91 ± 0.47	0.69 ± 0.01
Sport 16	Sandy Loam	75.21 ± 0.4	12.26 ± 0.88	12.53 ± 0.03	78 ± 0.71	7.51 ± 0.28	128.50 ± 3.29	91.40 ± 1.68	1.70 ± 0.88	0.28 ± 0.01	0.17 ± 0.02	0.97 ± 0.03	18.91 ± 0.37	0.66 ± 0.06
Spot 17	Sandy Loam	65.14 ± 0.1	20.15 ± 0.7	14.71 ± 0.37	90 ± 0.06	7.77 ± 0.75	159.40 ± 4.72	113 ± 1.93	4.64 ± 0.93	0.16 ± 0.01	0.20 ± 0.01	1.83 ± 0.04	19.89 ± 1.38	0.86 ± 0.04
Spot 18	Loam	43.28 ± 0.05	44.21 ± 0.75	12.51 ± 0.47	95 ± 0.09	8.18 ± 0.18	181.60 ± 5.61	129 ± 2.92	3.26 ± 0.61	0.12 ± 0.01	0.21 ± 0.03	4.14 ± 0.06	82.02 ± 0.91	0.90 ± 0.06
Spot 19	Sandy Loam	55.04 ± 0.05	28.32 ± 0.1	16.64 ± 0.2	84 ± 0.28	7.98 ± 0.34	178.15 ± 2.76	126.50 ± 1.73	5.10 ± 0.28	0.32 ± 0.04	0.11 ± 0.01	2.69 ± 0.19	20.09 ± 0.73	0.65 ± 0.03
Spot 20	Sandy Loam	62.39 ± 1.2	29.31 ± 0.3	8.3 ± 0.24	95 ± 0.02	7.33 ± 0.16	192.10 ± 5.82	139.50 ± 2.84	1.01 ± 0.16	0.38 ± 0.01	0.08 ± 0.01	2.95 ± 0.32	3.88 ± 0.13	0.98 ± 0.09
Spot 21	Sandy Loam	67.45 ± 2.1	26.01 ± 0.6	6.54 ± 2.48	80 ± 0.38	7.35 ± 0.27	189.05 ± 2.61	134.50 ± 3.49	5.50 ± 0.95	0.27 ± 0.01	0.23 ± 0.01	2.30 ± 0.71	5.83 ± 0.17	0.80 ± 0.07
Spot 22	Loam	44.67 ± 1.25	42.18 ± 0.7	13.15 ± 0	48 ± 0.12	5.96 ± 0.12	44.35 ± 1.96	31.65 ± 1.83	4.17 ± 0.82	0.29 ± 0.02	0.20 ± 0.03	6.04 ± 0.34	1.18 ± 0.24	1.29 ± 0.04
Spot 23	Sandy Loam	67.14 ± 1.85	22.51 ± 0.85	10.35 ± 0.01	59 ± 0.27	7.25 ± 0.28	157.85 ± 2.87	112 ± 1.73	5.38 ± 0.73	0.31 ± 0.01	0.16 ± 0.04	3.06 ± 0.91	6.00 ± 0.19	1.03 ± 0.12
Spot 24	Sandy Loam	65.43 ± 0.9	27.06 ± 0.45	7.51 ± 0.01	87 ± 0.17	6.59 ± 0.36	99.95 ± 1.86	71.10 ± 0.95	4.76 ± 0.34	0.30 ± 0.06	0.12 ± 0.01	3.23 ± 0.71	1.72 ± 0.47	1.13 ± 0.19
Spot 25	Sandy Loam	63.11 ± 1.15	29.26 ± 0.8	7.63 ± 0.01	80 ± 0.04	6.88 ± 0.49	225.50 ± 6.74	158 ± 1.64	5.66 ± 0.73	0.54 ± 0.05	0.16 ± 0.07	1.47 ± 0.02	3.02 ± 0.34	0.49 ± 0.02
Spot 26	Loam	48.26 ± 4.4	34.23 ± 0.85	17.51 ± 0.46	80 ± 0.13	6.71 ± 0.24	149.05 ± 5.31	106 ± 2.59	5.61 ± 0.37	0.18 ± 0.01	0.18 ± 0.09	3.36 ± 0.09	1.21 ± 0.21	0.61 ± 0.08
Spot 27	Sandy Loam	71.19 ± 5.95	17.34 ± 1.1	11.49 ± 0.34	75 ± 0.19	7.48 ± 0.37	163.80 ± 3.89	118.5 ± 1.82	5.38 ± 0.84	0.41 ± 0.01	0.19 ± 0.04	3.49 ± 0.14	1.71 ± 0.39	0.66 ± 0.05
Spot 28	Sandy Clay Loam	66.18 ± 1.25	26.01 ± 1.1	7.82 ± 2.98	79 ± 0.28	7.65 ± 0.29	185.40 ± 2.73	131.5 ± 2.85	6.55 ± 0.27	0.65 ± 0.07	0.22 ± 0.01	3.66 ± 0.18	4.23 ± 0.64	1.12 ± 0.13
Spot 29	Loam fine Sand	79.06 ± 0.8	5.34 ± 0.85	15.6 ± 1.57	61 ± 0.18	6.82 ± 0.18	201 ± 2.94	142.5 ± 4.72	6.15 ± 0.49	0.39 ± 0.02	0.14 ± 0.02	1.45 ± 0.02	2.45 ± 0.53	1.22 ± 0.16
Spot 30	Sandy Loam	65.47 ± 0.75	7.92 ± 0.6	26.52 ± 3.13	83 ± 0.14	7.40 ± 0.62	151.05 ± 3.73	108.5 ± 1.49	5.18 ± 0.75	0.45 ± 0.01	0.17 ± 0.02	1.93 ± 0.34	7.73 ± 0.29	0.85 ± 0.01
Spot 31	Sandy Loam	69.41 ± 0.5	9.29 ± 1.25	21.29 ± 0.82	90 ± 0.13	7.46 ± 0.76	38.95 ± 1.64	27.7 ± 1.67	5.38 ± 0.47	0.34 ± 0.02	0.10 ± 0.01	2.41 ± 0.03	2.30 ± 0.14	1.27 ± 0.08
Spot 32	Sandy Loam	61.05 ± 0.05	12.52 ± 0.8	26.41 ± 2.57	95 ± 0.03	5.96 ± 0.43	65.55 ± 1.98	46.3 ± 2.48	5.46 ± 0.87	0.48 ± 0.01	0.21 ± 0.03	3.32 ± 0.42	5.13 ± 0.67	1.40 ± 0.09
Spot 33	Sandy Loam	72.58 ± 0.2	9.84 ± 0.85	17.59 ± 1.09	86 ± 0.24	6.73 ± 0.28	27.30 ± 0.85	18.15 ± 1.37	5.54 ± 0.99	0.69 ± 0.04	0.09 ± 0.01	2.79 ± 0.34	1.74 ± 0.09	0.69 ± 0.04
Spot 34	Sandy Loam	64.19 ± 1.7	8.21 ± 0.3	27.61 ± 3.9	83 ± 0.03	7.11 ± 0.56	73.30 ± 1.49	51.45 ± 1.89	6.39 ± 0.92	0.56 ± 0.03	0.16 ± 0.01	5.88 ± 0.72	1.62 ± 0.18	0.88 ± 0.01
Spot 35	Sandy Loam	68.34 ± 0.45	13.67 ± 0.35	18.06 ± 2.9	79 ± 0.23	6.65 ± 0.82	58.10 ± 0.49	40.55 ± 1.58	5.22 ± 0.85	0.55 ± 0.04	0.22 ± 0.03	2.87 ± 0.21	4.34 ± 0.26	1.45 ± 0.18
Spot 36	Sandy Loam	68.54 ± 1.25	15.26 ± 2.6	16.27 ± 0.25	59 ± 0.25	7.87 ± 0.68	258 ± 1.38	166.5 ± 3.49	5.77 ± 0.73	0.84 ± 0.08	0.28 ± 0.17	2.19 ± 0.31	6.61 ± 0.28	1.16 ± 0.31
Spot 37	Sandy Loam	74.58 ± 1.65	8.98 ± 1.75	16.49 ± 0.41	62 ± 0.2	7.78 ± 0.41	85.85 ± 0.99	60.85 ± 2.32	4.87 ± 0.12	0.85 ± 0.02	0.13 ± 0.04	1.41 ± 0.01	3.18 ± 0.92	0.93 ± 0.05
Spot 38	Sandy Loam	71.39 ± 0.55	11.68 ± 1.75	16.83 ± 1.24	75 ± 0.65	8.17 ± 0.53	136.4 ± 2.98	96.15 ± 2.47	4.87 ± 0.24	0.74 ± 0.03	0.19 ± 0.06	2.11 ± 0.34	2.88 ± 0.12	1.09 ± 0.02
Spot 39	Sandy Loam	68.53 ± 0.4	14.52 ± 2.01	18 ± 0.05	62 ± 0.85	7.81 ± 0.24	124.45 ± 1.46	86.9 ± 1.86	5.77 ± 0.65	1.17 ± 0.01	0.12 ± 0.02	1.31 ± 0.24	3.02 ± 0.41	1.00 ± 0.01
Spot 40	Sandy Loam	70.67 ± 0.75	16.49 ± 0.75	12.86 ± 0.84	65 ± 0.62	7.43 ± 0.73	73.4 ± 0.92	51.7 ± 1.12	4.36 ± 0.34	0.51 ± 0.01	0.13 ± 0.01	1.54 ± 0.08	1.89 ± 0.57	1.15 ± 0.06
Spot 41	Loamy Fine Sand	80.19 ± 1.35	12.09 ± 0.04	7.69 ± 0.42	58 ± 0.4	7.31 ± 0.32	130.15 ± 2.82	92.35 ± 1.47	7.15 ± 0.47	1.27 ± 0.04	0.2 ± 0.01	1.85 ± 0.07	4.89 ± 0.27	1.04 ± 0.04
Spot 42	Sandy Loam	65.07 ± 0.8	16.28 ± 0.01	18.65 ± 0.87	78 ± 0.85	8.23 ± 0.29	126.8 ± 3.48	90.15 ± 2.47	4.5 ± 0.82	0.61 ± 0.01	0.23 ± 0.03	3.29 ± 0.35	3.40 ± 0.31	1.01 ± 0.02
Spot 43	Sandy Loam	75.07 ± 0.23	9.44 ± 0.33	15.49 ± 0.13	81 ± 0.2	7.78 ± 0.49	83.4 ± 1.91	57.85 ± 1.37	4.4 ± 0.19	1.02 ± 0.01	0.21 ± 0.07	1.43 ± 0.12	3.24 ± 0.18	1.08 ± 0.03
Spot 44	Sandy Loam	73.15 ± 0.15	11.69 ± 0.41	15.24 ± 3.07	79 ± 0.5	7.26 ± 0.12	163.15 ± 3.64	115.5 ± 3.64	5.25 ± 0.41	0.57 ± 0.02	0.26 ± 0.01	1.64 ± 0.31	12.46 ± 0.91	0.86 ± 0.11

The distribution and abundance of 8 earthworm species w.r.t. range of physico-chemical properties of soil at different sampling sites is given in Table 3. The range of physico-chemical properties of soil for *Polypheretima elongata,* and *Perionyx excavatus* is not given due to their presence at a single site only. The *M. posthuma, A. morrisi, A. alexandri, E. incommodus* and *M. birmanica* were present in slightly alkaline soil while *E. waltoni, M. houlleti, and O. beatrix* were present from slightly acidic to slightly alkaline soil. The organic carbon (%) range for *M. posthuma* (OC from 1.45 to 9.53) was high as compared to other species. High range of N (0.12–2.68 g/Kg) and P (0.06–0.26 g/Kg) content was also observed at *M. posthuma* sampling sites which is due to the use of nitrogen and phosphorus in the form of fertilizers in the cultivated fields.

**Table 3:** The range of physico-chemical properties of soil for different earthworm species

S. no.	Earthworm species*	Sand (%)	Silt (%)	Clay (%)	Moisture (%)	pH	EC (µS)	TDS (mg/L)	OC (%)	N (g/Kg)	P (g/Kg)	K (g/Kg)	Ca (g/Kg)	Na (g/Kg)
1.	*M. posthuma*	43.28–80.19	9.44–44.21	6.54–20.75	76–97	7.15–8.57	38.6–181.6	27.45–166.5	1.45–9.53	0.12–2.68	0.06–0.26	0.97–4.14	1.89–107.34	0.44–1.09
2.	*A. morrisi*	64.19–71.19	8.22–20.31	10.20–27.59	65–89	7.11–7.98	73.3–118.15	51.45–83.95	3.08–6.39	0.51–1.05	0.08–0.16	1.54–5.88	1.62–10.20	0.75–1.15
3.	*A. alexandri*	55.04–74.58	8.93–28.32	12.86–16.64	62–90	7.43–7.98	73.4–178.15	51.7–125.5	4.36–5.10	0.16–0.85	0.11–0.20	1.41–2.69	1.89–20.9	0.65–1.15
4.	*E. waltoni*	44.67–79.06	5.34–42.18	11.47–18.05	48–93	5.96–8.65	44.35–201	31.65–142.5	2.77–6.15	0.18–1.31	0.10–0.22	0.98–6.04	1.18–16.91	0.61–1.45
5.	*E. incommodus*	45.14–81.47	8.46–38.91	10.07–15.95	79–84	7.14–8.65	80.55–897.5	57.45–89.8	2.77–3.70	1.31–1.35	0.21–0.22	2.38–7.45	8.67–18.64	0.74–0.94
6.	*M. houlleti*	53.14–67.45	7.92–32.41	7.51–26.61	80–95	5.96–7.40	38.60–225.5	27.45–158	3.83–5.66	0.26–0.54	0.06–0.23	1.47–3.32	1.72–12.92	0.49–1.40
7.	*O. beatrix*	44.67–72.58	8.22–42.18	6.54–27.59	48–95	5.96–7.65	27.30–192.10	18.15–139.5	1.01–6.55	0.18–0.69	0.08–0.23	2.30–6.04	1.18–6.0	0.61–1.29
8.	*M. birmanica*	67.14–80.19	12.09–22.51	7.69–10.35	58–59	7.25–7.31	130.15–157.85	92.35–112	5.38–7.15	0.31–1.27	0.16–0.20	1.85–3.06	4.89–6.0	1.03–1.04

* The physico–chemical properties range for *Polypheretima elongata* and *Perionyx excavatus* is not given due to their presence at single site only

### Impact of soil properties on earthworm abundance

**Fig. 2:**
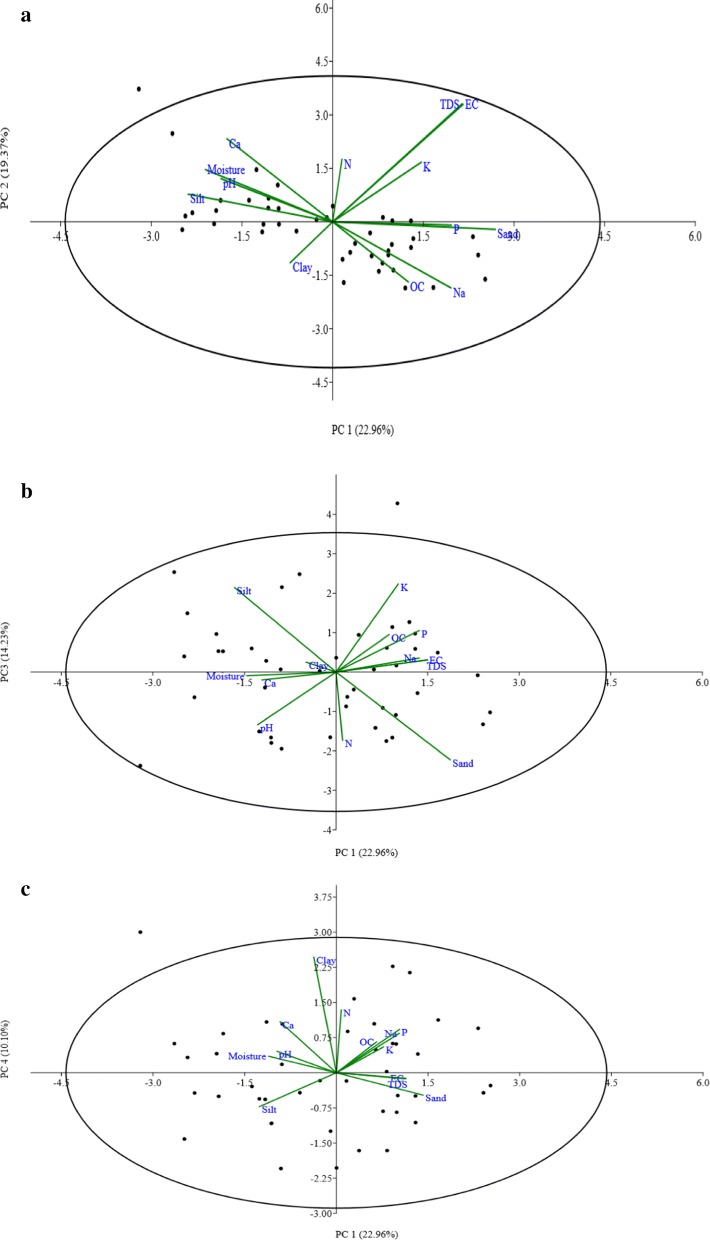
Biplot of PCA of 13 different physico-chemical properties of soil, **a** PC1 vs PC2; **b** PC1 vs PC3; **c** PC1 vs PC4. The PC1, PC2, PC3 and PC4 explain 22.96%, 19.37%, 14.23% and 10.10% of variance respectively in datasets. The components with variance less than 10% were excluded. The Varimax rotation with Kaiser Normalization method was used for the extraction of principal components

The Principal component analysis (PCA) was used on 13 different variables of soil for 44 different sites to study the influence of soil properties on the distribution and abundance of earthworm species. The PCA analysis gives four different principal components (PC) i.e. PC1, PC2, PC3 and PC4 which causing variance (%) of 22.96, 19.37, 14.23 and 10.10 respectively (Table 4). As indicated by Liu et al. [18], the factors were considered weak, moderate and strong when absolute loading values were < 0.50, 0.50–0.75 and > 0.75 respectively. The variance in PC1 is due to pH, EC, TDS, and K; in PC2 is due to sand, clay and silt; in PC3 is due to Moisture and OC; in PC4 is due to Ca, Na, P and N. The first two components of the PCA i.e. PC1 (22.96%) and PC2 (19.37%) of physico-chemical variables account for 42.33% of total variance with positive strong loading of pH, EC, TDS and K in PC1 while strong negative loading of clay and strong positive loading of silt and sand in PC2 (Fig. 2a). On the other hand, PC1 (22.96%) and PC3 (14.23%) account for 37.19% of total variance with strong positive loading of OC and strong negative loading of moisture in PC3 (Fig. 2b). The principal component PC4 accounts for the variance of 10.10% with positive loading of Ca, N, Na & P (Fig. 2c). The earthworm abundance and soil properties have significant direct relation (PERMANOVA, F = 22.1, P < 0.05; Mantel test, R = 0.14, P < 0.05) and soil properties also favours the earthworm abundance at a particular site and vice versa.

**Table 4:** The eigenvalues and principal components of soil variables along with variances in percentage

Variables	PC1	PC2	PC3	PC4
Sand	0.127	*0.933*	– 0.006	0.118
Silt	– 0.047	*0.954*	– 0.076	– 0.105
Clay	– 0.167	– *0.898*	0.169	– 0.031
Moisture	0.085	0.174	– *0.705*	– 0.187
pH	*0.748*	0.113	– 0.010	– 0.272
EC	*0.943*	– 0.167	– 0.005	– 0.036
TDS	*0.944*	– 0.165	– 0.010	– 0.043
OC	– 0.009	– 0.039	*0.962*	0.137
Ca	0.164	0.345	– 0.267	*0.614*
Na	– 0.097	– 0.193	– 0.029	*0.802*
K	*0.735*	0.295	– 0.022	0.248
P	0.282	0.069	0.274	*0.660*
N	0.147	– 0.247	– 0.177	*0.768*
Earthworms abundance	– 0.378	0.427	0.390	– 0.149
Eigenvalue	3.215	2.712	1.992	1.414
Variance (%)	22.96	19.37	14.23	10.10
Cumulative variance (%)	22.96	42.33	56.57	66.67

Extraction method: principal component analysis. rotation method: varimax with kaiser normalization

## Discussion

### Effect of land use pattern on earthworm diversity

The abiotic factors, vegetation type and physico-chemical properties of soil determined the abundance of earthworms [1]. *Metaphire posthuma* was observed at maximum sampling sites including both cultivated and non-cultivated while *Polypheretima elongata* and *Perionyx excavatus* were restricted to a single site, least abundant and reported from non-cultivated sites. Thus*, M. posthuma* was found to be the most stable and adapted earthworm species in cultivated land use which corroborated with the findings of Singh et al. [1] who also observed the same results. The high abundance of *M. posthuma* at cultivated sites might be due to their endogeic ecological nature [1]. Mariotte et al. [19] also reported that earthworm species with endogeic ecological category were least affected as compare to epigeic and anecic earthworm species. On the other hand, ploughing damages the burrows of earthworm in the soil which directly disturbs anecic and epigeic earthworm species as compared to species with the endogeic ecological category. This might be the reason for the low abundance and diversity of anecic and epigeic species in agricultural fields as compared to gardens and nurseries [20]. On the other hand, in our study other endogeic earthworm species viz *M. houlleti, O. beatrix, M. birmanica* and *Polypheretima elongata* were also reported but their abundance is much less in cultivated fields as compare to non-cultivated fields. The reason for this may be due to agricultural practices. The more abundance of earthworm species was observed at margins of the paddy fields but no earthworm was reported inside fields because paddy cultivation requires intensive ploughing and water. This might be the reason for the abundance of earthworm at field margins. Thus, our study is corroborated with Frazão et al. [3] and van Schaik et al. [17] who also observed high density and species richness of earthworm in field margins as compared to the fields having wheat cultivation.

In the present study, the earthworm diversity indices were changed as the agro-ecosystem changed from cultivated to non-cultivated pattern. The highest values of diversity indices were observed in non-cultivated sites as compared to cultivated sites which were due to the availability of only one species of earthworm i.e. *M. posthuma* in cultivated sites as compare to non-cultivated sites. Solomou et al., [21] also observed that the diversity indices change from non-cultivated to cultivated agro-ecosystem. The agricultural management practices such as deep ploughing, fertilizer and pesticide application directly affect the earthworm species present within the soil which usually affects the diversity indices. Bartz et al. [22] also observed that minimum ploughing has greater earthworm richness as compared to the site with conventional tillage. Margerie et al. [23] and Goswami [24] also supported our observation that diversity indices usually change from one sampling site to another due to changes in the habitat.

### Soil variables and earthworm abundance

The physico-chemical properties of soil directly affect the earthworm abundance and hence diversity indices. In the present study, high contents of N, P and TDS were reported in the cultivated fields and these cultivated fields have also less earthworm diversity with restriction to single earthworm species. We have also observed that sites having the application of cattle dung as organic manure like in gardens have more earthworm species than agriculture land. The input of organic manures and non-conventional farming system also improves soil quality which promotes earthworm presence within the soil [21, 25]. Singh et al. [26] also reported that the use of sheep dung in the intensive grassland management increase the earthworm population and their abundance and number increase up to four times which directly affected the earthworm diversity indices [27]. The present study also reported that agricultural management practices such as ploughing, tillage etc. also has an effect on the abundance of earthworms at a particular site.

The principal component analysis was also applied to the 13 different physico-chemcial variables which resulted in total of 4 principal components (PC) which explained 66.67% of the total variance. The PC1 explained 22.96% of total variance which was due to pH, EC, TDS and K. This validates the outcomes of Sanchez et al. [28] which explained that earthworm prefers soil having salt concentrations. The pH at a particular site is also an important factor for earthworm distribution as earthworms can survive only in neutral but also in slightly acidic to slightly alkaline soil conditions. Soil pH did not directly affect the abundance of earthworms at a particular site but indirectly pH drives other chemical processes in earthworms which affect nutrient availability [29]. In the present study, the pH of the soil varied from 5.96 to 8.6. The earthworm’s species respond very quickly to variations in pH at a particular site and they are generally avoiding soil having pH values less than 4.5, favoring pH between 5.0 and 7.4 [30]. McCallum et al. [31] also observed that diversity and abundance of earthworms are very low in soil having a pH near 4.5. Most of the studies reported that earthworms can tolerate a pH range of 5.0 to 8.0 and an abundance of earthworms increase as pH was shifted from acidic or basic to neutral. De Wandeler et al. [16] also observed that the earthworm’s abundance and diversity in soil increased with an increase in pH from acidic to neutral and maximum earthworm abundance was found near pH 7. The EC also plays a vital role in earthworm metabolism [32]. Thus, PC1 explained chemical factors for earthworm’s distributions. The PC2 explained 19.37% of total variance respectively which was due to sand, silt and clay. Yvan et al. [33] described that soil texture also influences the activity and growth of earthworm. Higher clay content in soil favours growth and abundance of earthworm’s species [34] which is clearly represented in our study. Thus, PC2 explained the soil texture factor. The PC3 explained 14.23% of total variance which was due to OC and moisture. According to Chan and Barchia [35], organic carbon is the critical factor for the earthworm distribution as it helps in determines the type and nature of food for an earthworm. Bartz et al. [22] and Jänsch et al. [36] also observed a significant relationship between soil OC and earthworm abundance. They observed that sites with high earthworm abundance have high soil OC content and vice versa. The presence of leaf litter in the soil also favors the earthworm population due to the easy availability of organic matter [37]. The Moisture is a critical factor for earthworm distribution due to the cutaneous mode of respiration [38]. Walsh and Johnson-Maynard [39] reported that earthworms were absent from the driest sites and their high density and biomass depend on local conditions like soil properties and management. Talavera et al. [40] have reported that both moisture and OC are the key factors for the prediction of earthworm communities at a particular site. Thus, PC3 explains the growth factor. The PC4 explained 10.10% of variance which was due to N, Ca, Na and P; which explain the edaphic factors for earthworm’s distributions. Thus, PCA explained that earthworm communities and their diversity indices are directly correlated with the physico-chemical characteristics of soil at the particular site.

## Conclusion

This study provides information about the pattern of earthworm diversity in cultivated and non-cultivated fields and how soil physico-chemical properties affect the earthworm diversity indices in different agro-ecosystem. It was concluded that cultivated fields having less earthworm diversity as compared to non-cultivated fields. The *M. posthuma* was observed at maximum sampling sites and found in all types of vegetation especially in the cultivated fields having paddy plantation while others endogeic earthworm species were absent in paddy plantation. The change in physico-chemical properties of soil also alters the earthworm diversity indices. The principal component analysis also showed that the physico-chemical properties of soil play a critical role in earthworm distribution. The diversity indices also change due to the conventional farming system which directly affects the earthworm abundance. The farmers should be aware of the roles of earthworms in soil and also must be encouraged to shift their agricultural practices from conventional to organic. These practices not only increase earthworm diversity and abundance but also helps to maintain soil enriched with various types of major and macro-nutrients.

## Methods

### Site study

**Fig. 3:**
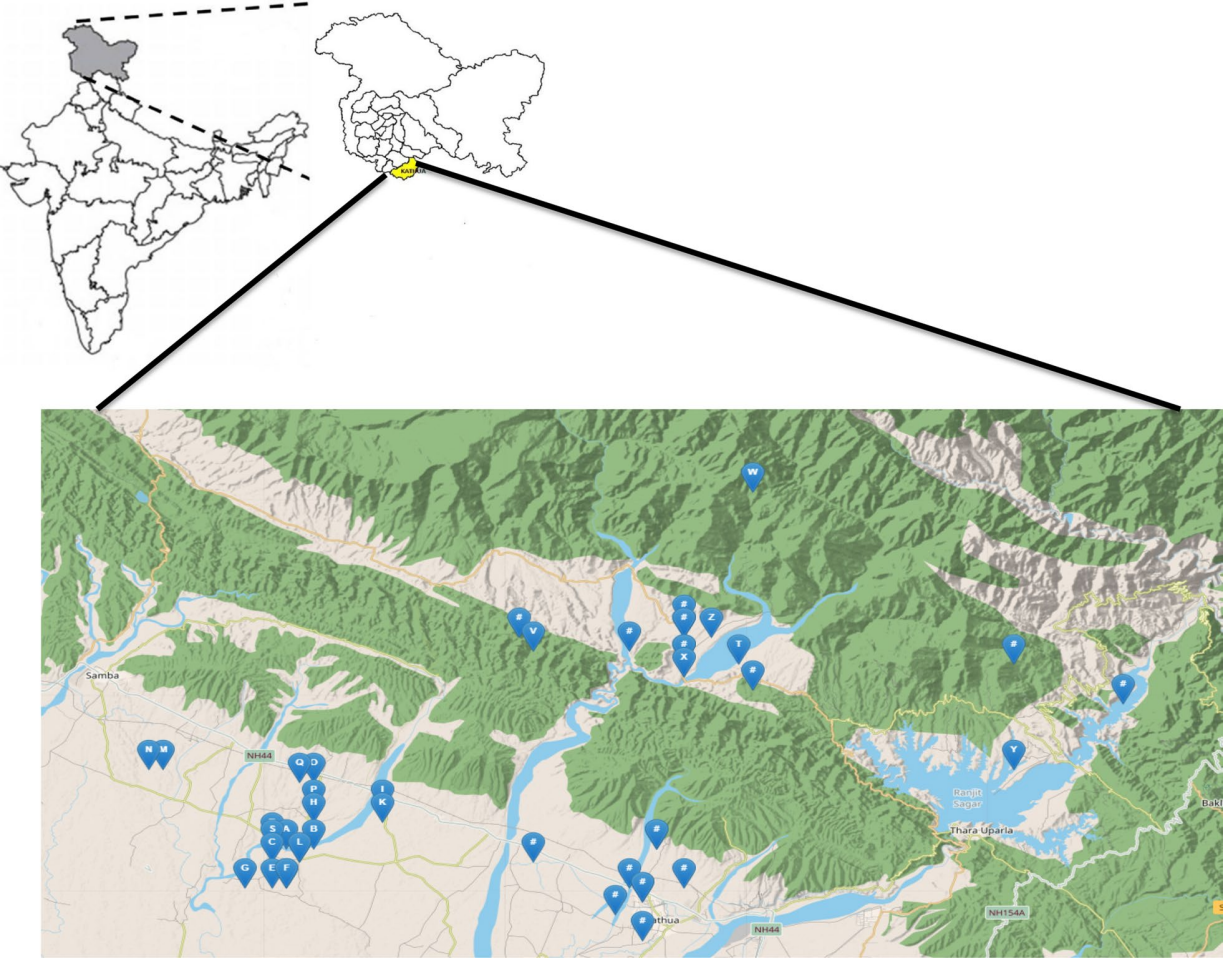
Map showing the location of study sites

The earthworm sampling was done during two consecutive years in monsoon and post-monsoon seasons of the year 2015 and 2016 in the district Kathua (Jammu & Kashmir, India) (Fig. 3). This monsoon and post-monsoon period were chosen for sampling due to easy availability and high maturity of earthworms. The Kathua district is situated in 32°34′ N 75°29′ E with annual rainfall in this district is approximately 1672 mm. The summer temperature arises as high as 48 °C in the plains and in winter temperature touches to sub-zero mainly in upper hilly areas. The area under Kathua district experiences a wide range of climate from subtropical to temperate areas. The major crop of the study site is paddy, wheat, barley, and maize.

### Earthworm sampling and Identification

Earthworm’s sampling was done at 44 sites having cultivated and non-cultivated agro-ecosystem which were chosen randomly (Additional file 1: Table S1). The collections of earthworms were mainly done during their activation period i.e. morning by using hand sorting (1 m × 1 m area) followed by AITC extraction solution [15] to collect deep burrowing earthworm species. The collected earthworms from all the diverse sites with a reasonable amount of soil were placed in plastic bags, named with the site name, sampling date, etc. The earthworms were washed with tap water and narcotized with 70% ethyl alcohol. The narcotized earthworms were transferred to the flat tray with a 5% formalin solution in a straight position for 4–6 h followed by preservation in test tubes containing 5% formalin. All the test tubes were labeled with the place of collection, date of collection and their habitat. The earthworms were identified according to the keys provided by Julka [41].

### Physico-chemical analysis of soil

The soil samples were analyzed for texture, total dissolved solids (TDS), pH, electrical conductivity (EC), organic carbon (OC), nitrogen (N), sodium (Na), calcium (Ca), phosphorus (P), potassium (K). The method of Bouyoucos [42] as used for soil texture analysis. The content of pH, EC, and TDS was analyzed by making the suspension of 1:10 (soil sample: distilled water) using a shaker and finally measured with a digital meter (Eutech Instruments). The OC was measured by using Nelson and Sommers [43] method after igniting the soil samples in a muffle furnace at 550 °C. The N was analyzed according to Bremner and Mulvaney [44] method after digesting the soil sample with concentrated H_2_SO_4_ followed by running the sample in Kjeldahl assembly and finally titration was done with 0.01 N HCl. The process of John [45] was used for phosphorus estimation after digesting the soil with 1:4 of perchloric acid and nitric acid respectively followed by using a spectrophotometer (Systronics). The content of Na, K and Ca was analyzed from samples digested for phosphorus by using Flame Photometer-128 (Systronics).

### Statistical analysis

The data for physico-chemical properties of the soil is represented as mean ± S.E. of triplicate data. The Shannon-Wiener index, species richness index and species evenness for each sampling site were also calculated by using standard calculation as suggested by Shannon and Wiener [46], Margalef [47] and Pielou [48] respectively. Principal Component Analysis (PCA) was used to know the major components in the soil with one or more variable which affects the diversity of earthworms at a particular site. PERMANOVA and Mantel test were also applied to test the relationship between soil variables and earthworm abundance by using similarity matrices and the resemblance between the matrices was done by using Bray–Curtis similarity measures with 9999 random permutations. The past statistical software (version 3) and SPSS 16 (version 21) programme were used for the statistical analysis of the data.

## Supplementary information

**Additional file 1: Table S1.** The different sampling sites along with their agroecosystem, vegetation and GPS coordinates.

## Data Availability

The data sets used and/or interpreted during the present research are accessible from the Corresponding author on reasonable request.

## Abbreviations

PCA: Principal component analysis
PC: Principal component
PERMANOVA: Permutational multivariate analysis of variance
S.E: Standard error
AITC: Allyl isothiocyanate
TDS: Total dissolved solids
EC: Electrical conductivity
OC: Organic carbon
N: Nitrogen
Na: Sodium
Ca: Calcium
P: Phosphorus
K: Potassium
HCl: Hydrochloric acid
H_2_SO_4_: Sulphuric acid
H’: Shannon-Wiener Diversity Index
D_Mg_: Margalef species richness index
E: Pielou species evenness
*Mp*: *Metaphire posthuma*
*Ew*: *Eutyphoeus waltoni*
*Ei*: *Eutyphoeus incommodus*
*Am*: *Amynthas morrisi*
*Mh*: *Metaphire houlleti*
*Aa*: *Amynthas alexandri*
*Ob*: *Octochaetona beatrix*
*Mb*: *Metaphire birmanica*
*Pex*: *Perionyx excavatus*
*Pe*: *Polypheretima elongata*
