# Supplementary material for: Soil properties changes earthworm diversity indices in different agro-ecosystem
Source: BMC Ecol. 2020 May 7;20:27. doi: 10.1186/s12898-020-00296-5 (PMC7203807; doi:10.1186/s12898-020-00296-5)
Supplement: Supplementary file 1 — Additional file 1: Table S1. The different sampling sites along with their agroecosystem, vegetation and GPS coordinates. [file 12898_2020_296_MOESM1_ESM.doc]

**Additional file 1: Table S1:** The different sampling sites along with their agroecosystem, vegetation and GPS

coordinates.

| **Spot No.** | **Agroecosystem** | **Vegetation** | **GPS Coordinates** |
| --- | --- | --- | --- |
| Spot 1 | Non-cultivated | *Citrus sinensis* (Orange) and *Ocimum sanctum* (Tulsi) | 32.438819, 75.251581 |
| Spot 2 | Non-cultivated | [*Parthenium hysterophorus*](https://en.wikipedia.org/wiki/Parthenium_hysterophorus) (Congress grass) | 32.436049, 75.272643 |
| Spot 3 | Non-cultivated | Grass | 32.423433, 75.248170 |
| Spot 4 | Non-cultivated | Flowers with grasses | 32.402337, 75.250168 |
| Spot 5 | Cultivated | *Brassica nigra* (Mustard) | 32.404149, 75.248022 |
| Spot 6 | Cultivated | *Abelmoschus esculentus* (Ladyfinger) | 32.404004, 75.252056 |
| Spot 7 | Cultivated | *Saccharum officinarum* (Sugarcane) and Grass | 32.401793, 75.226830 |
| Sport 8 | Non-cultivated | *Mangifera indica* (Mango) and grasses | 32.457063, 75.273156 |
| Spot 9 | Non-cultivated | [*Parthenium hysterophorus*](https://en.wikipedia.org/wiki/Parthenium_hysterophorus) (Congress grass) and grass | 32.467276, 75.327705 |
| Spot 10 | Non-cultivated | *Mangifera indica* | 32.422879, 75.265702 |
| Spot 11 | Cultivated | *Pennisetum glaucum* (Bajra) | 32.459786, 75.325337 |
| Spot 12 | Cultivated | *Pennisetum glaucum* | 32.422527, 75.262175 |
| Sport 13 | Cultivated | *Pennisetum glaucum* | 32.495281, 75.161129 |
| Spot 14 | Cultivated | *Saccharum officinarum* | 32.498710, 75.159586 |
| Spot 15 | Cultivated | *Pennisetum glaucum* and [*Parthenium hysterophorus*](https://en.wikipedia.org/wiki/Parthenium_hysterophorus) | 32.482109, 75.275392 |
| Sport 16 | Non-cultivated | *Mangifera indica* | 32.469727, 75.272729 |
| Spot 17 | Non-cultivated | Grass | 32.487396, 75.265521 |
| Spot 18 | Cultivated | *Oryza sativa* (Paddy), | 32.435109, 75.248268 |
| Spot 19 | Cultivated | *Saccharum officinarum* | 32.437644, 75.244750 |
| Spot 20 | Non-cultivated | Grass | 32.570865, 75.586731 |
| Spot 21 | Non-cultivated | Garden | 32.579942, 75.546776 |
| Spot 22 | Cultivated | Fodder | 32.587567, 75.430667 |
| Spot 23 | Non-cultivated | [*Parthenium hysterophorus*](https://en.wikipedia.org/wiki/Parthenium_hysterophorus) | 32.700865, 75.599873 |
| Spot 24 | Non-cultivated | *Lantana camara* | 32.564345, 75.543270 |
| Spot 25 | Non-cultivated | Grass | 32.496709, 75.780718 |
| Spot 26 | Non-cultivated | Garden | 32.593544, 75.567875 |
| Spot 27 | Cultivated | Vegetables fields having cattle dung application | 32.570299, 75.781347 |
| Spot 28 | Non-cultivated | Grass | 32.582179, 75.503358 |
| Spot 29 | Cultivated | *Abelmoschus esculentus* | 32.579334, 75.545354 |
| Spot 30 | Non-cultivated | Grass | 32.558373, 75.592459 |
| Spot 31 | Non-cultivated | [*Parthenium hysterophorus*](https://en.wikipedia.org/wiki/Parthenium_hysterophorus) | 32.547161, 75.860037 |
| Spot 32 | Cultivated | *Oryza sativa* (Paddy) | 32.604450, 75.540592 |
| Spot 33 | Non-cultivated | *Murraya koenigii* | 32.598190, 75.423530 |
| Spot 34 | Non-cultivated | *Mangifera indica* | 32.594498, 75.541713 |
| Spot 35 | Non-cultivated | Grass | 32.503289, 75.810963 |
| Spot 36 | Non-cultivated | Grass | 32.398088, 75.517127 |
| Spot 37 | Non-cultivated | Rose plantation with Grass | 32.425457, 75.439382 |
| Spot 38 | Cultivated | *Oryza sativa* (Paddy) | 32.367645, 75.515065 |
| Spot 39 | Cultivated | *Cucumis sativus* (Cucumber) | 32.386128, 75.499015 |
| Spot 40 | Non-cultivated | Grass | 32.434086, 75.526626 |
| Spot 41 | Non-cultivated | Grass | 32.402725, 75.501590 |
| Spot 42 | Cultivated | *Oryza sativa* (Paddy) | 32.397556, 75.510805 |
| Spot 43 | Cultivated | *Oryza sativa* (Paddy) | 32.431949, 75.525596 |
| Spot 44 | Cultivated | *Oryza sativa* (Paddy) | 32.408219, 75.540995 |
